# Genomic Analyses Reveal Evidence of Independent Evolution, Demographic History, and Extreme Environment Adaptation of Tibetan Plateau *Agaricus bisporus*

**DOI:** 10.3389/fmicb.2019.01786

**Published:** 2019-08-13

**Authors:** Lei Sun, Yuhua Fu, Yang Yang, Xinxin Wang, Weijie Cui, Dan Li, Xiaohui Yuan, Zhiwu Zhang, Yongping Fu, Yu Li

**Affiliations:** ^1^Engineering Research Center of Chinese Ministry of Education for Edible and Medicinal Fungi, Jilin Agricultural University, Changchun, China; ^2^School of Computer Science and Technology, Wuhan University of Technology, Wuhan, China; ^3^Department of Crop and Soil Sciences, College of Agricultural, Human, and Natural Resource Sciences, Washington State University, Pullman, WA, United States

**Keywords:** button mushroom, genome sequencing, demographic history, adaptation, resequencing

## Abstract

*Agaricus bisporus* distributed in the Tibetan Plateau of China has high-stress resistance that is valuable for breeding improvements. However, its evolutionary history, specialization, and adaptation to the extreme Tibetan Plateau environment are largely unknown. Here, we performed *de novo genome* sequencing of a representative Tibetan Plateau wild strain ABM and comparative genomic analysis with the reported European strain H97 and H39. The assembled ABM genome was 30.4 Mb in size, and comprised 8,562 protein-coding genes. The ABM genome shared highly conserved syntenic blocks and a few inversions with H97 and H39. The phylogenetic tree constructed by 1,276 single-copy orthologous genes in nine fungal species showed that the Tibetan Plateau and European *A. bisporus* diverged ∼5.5 million years ago. Population genomic analysis using genome resequencing of 29 strains revealed that the Tibetan Plateau population underwent significant differentiation from the European and American populations and evolved independently, and the global climate changes critically shaped the demographic history of the Tibetan Plateau population. Moreover, we identified key genes that are related to the cell wall and membrane system, and the development and defense systems regulated *A. bisporus* adapting to the harsh Tibetan Plateau environment. These findings highlight the value of genomic data in assessing the evolution and adaptation of mushrooms and will enhance future genetic improvements of *A. bisporus*.

## Introduction

*Agaricus bisporus* is one of the most widely cultivated edible mushrooms in the world. Its wild resources were first discovered in Europe, and then the American population was reported (Callac et al., 1993; Kerrigan et al., 1998). With continuous investigation and collection, the distribution of wild resources was reported in Canada, Israel, Morocco, and the central and western regions of Africa (Kerrigan, 1995; Kerrigan et al., 1995; Sobieralski et al., 2010; Mata et al., 2016; Rokni et al., 2016; Sonnenberg et al., 2017). In the 1970s, wild *A. bisporus* was first discovered in the Tibetan Plateau (Wang et al., 2012), which need to withstand harsh environments such as alpine, anoxic, and strong ultraviolet rays.

Zhang et al. (2017) also reported wild strains of *A. bisporus* distributed in the Qilian Mountains (Zhang et al., 2017). Previous research has focused on the evaluation of Tibetan Plateau wild germplasm. Compared to the European and American germplasm, wild Tibetan Plateau resources possess higher disease resistance and richer genetic diversity (Fu et al., 2016b). However, its evolution history, the molecular-level mechanisms associated with extreme environmental adaption, and the genetic differentiation between the different populations remain unknown.

Extreme environments such as that in the Tibetan plateau significantly affect the growth and development of various organisms. Understanding the genetic background of organisms adapting to extremely harsh environments will facilitate in germplasm utilization, breeding improvement, and wild resource conservation. In recent years, with the development of sequencing technology, genome sequencing and comparative and population genomic analyses have been widely used to reveal the adaptations of species living in extremely harsh environments (Liu et al., 2014; Yang et al., 2016; Zhou et al., 2016). For example, the adaptation of wild boar to the Tibetan Plateau region and its domestication mechanism was revealed using comparative analysis of the domesticated pigs and Tibetan Plateau wild boars (Li et al., 2013). The metabolic processes related to adaptation to the desert stress were identified such as fat and water metabolism by the genome sequencing and comparative genome analysis of Bactrian camel, dromedary, and alpaca (Wu et al., 2014). For the environmental adaptation in fungi, the genes encoding transmembrane transport of substances and helicase activity related to cold stress response were identified in adaptation to climate at the continental scale in mushroom *Suillus brevipes* based on a population genomic analysis (Branco et al., 2017). However, the evolution and the adaptation of edible mushrooms living in extreme environments are largely unknown.

In this study, we used PacBio Single Molecule, Real-Time (SMRT) to perform *de novo* genome sequencing of the monokaryon strain ABM from a wild brown strain collected from the Tibetan Plateau in China and compared with the two reported genomes of monokaryons from Europe. And then whole-genome resequencing was performed to population genetics studies. Our specific objectives were as follows: (1) to reveal the difference in genome evolution and genome structure of *A. bisporus* genomes, including ABM from the extreme Tibetan Plateau environment and H97 and H39 from Europe; (2) to identify the genetic diversity, population structure, and genetic differentiation based on the population genomic analysis; and (3) to investigate the demographic history of wild strains from the Tibetan Plateau and elucidate its molecular mechanism for adaptation to the extreme harsh environment. Our study serves as a good foundation for further creation of the high-yield, high-quality, and stress-resistant germplasm in breeding novel varieties of *A. bisporus*, and also assist in genome analysis for genome evolution and adaptation of other fungi.

## Materials and Methods

### *Agaricus bisporus* Strains

The 29 *A. bisporus* strains preserved in our center were used for genome sequencing (Table 1), including 14 wild strains from the Tibetan Plateau in China, 4 Chinese breeding cultivars, and 11 strains representing European and American population. Among them we selected the wild strain AB58 from the Tibetan Plateau to perform *de novo* genome sequencing. According to our previous studies, ABM only has two basidiospores on each basidium by microscopic observations, indicating it was *A. bisporus* var. *bisporus*. The monokaryon strain ABM was isolated from AB58 using a protoplast-derived method: the dikaryotic mycelia were inoculated on PDA medium and cultured at 25°C for 10 days in the dark; 300 mg of mycelia were collected, and 2% Wall enzyme (Guangdong Institute of Microbiology, China) was added to enzymatic hydrolysis for 4 h under 30°C; using protoplast regeneration, nuclei staining by 4′,6-diamidino-2-phenylindole (DAPI, Sigma–Aldrich, United States) and microscopy identification, a monokaryon ABM was obtained. The ABM mycelia were then cultured on PDA medium with cellophane at 25°C for 15 days. Genome DNA of ABM was extracted using a high-efficiency genome extraction kit (CWBIO, China) and subjected to 0.8% agarose gel electrophoresis, NanoDrop 2000, and Qubit to detect genome integrity, purity, and concentration, respectively.

**TABLE 1:** Strains of *Agaricus bisporus* var. *bisporus* used for genome resequencing.

**Preservation number**	**Strain name**	**Original reference**	**Origin**	**Strain types**
CCMJ1009	AB3	A15	America	Cultivated strain
CCMJ1013	AB7	As2796	Fujian, China	Cultivated strain
CCMJ1018	AB12	As4580	Fujian, China	Cultivated strain
CCMJ1020	AB14	ZA	Germany	Cultivated strain
CCMJ1021	AB25	S130A	America	Cultivated strain
CCMJ1028	AB22	S46	Fujian, China	Cultivated strain
CCMJ1033	AB27	C13	America	Cultivated strain
CCMJ1035	AB29	72	America	Cultivated strain
CCMJ1037	AB31	U1	Netherlands	Cultivated strain
CCMJ1038	AB32	PSU310	America	Cultivated strain
CCMJ1039	AB33	126	Netherlands	Cultivated strain
CCMJ1053	AB35	M-1	Spain	Cultivated strain
CCMJ1106	AB43	2094	Tibet, China	Wild stain
CCMJ1109	AB42	Ag23	England	Cultivated strain
CCMJ1352 CCMJ1343	AB51 AB39	A12 W192	America Fujian, China	Cultivated strain Cultivated strain
CCMJ1347	AB54	T12387	Yunnan, China	Wild stain
CCMJ1350	AB57	W1	Sichuan, China	Wild stain
CCMJ1351	AB58	W2	Sichuan, China	Wild stain
CCMJ1360	AB67	W3	Sichuan, China	Wild stain
CCMJ1361	AB68	W4	Sichuan, China	Wild stain
CCMJ1363	AB70	W5	Sichuan, China	Wild stain
CCMJ1369	AB76	W6	Sichuan, China	Wild stain
CCMJ1372	AB79	W7	Sichuan, China	Wild stain
CCMJ1374	AB81	W11	Sichuan, China	Wild stain
CCMJ1377	AB84	W8	Sichuan, China	Wild stain
CCMJ1379	AB86	W12	Sichuan, China	Wild strain
CCMJ1381	AB88	W9	Sichuan, China	Wild stain
CCMJ1384	AB91	W10	Sichuan, China	Wild stain

### *De novo* Genome Sequencing of *A. bisporus* Collected From the Tibetan Plateau in China

*De novo* genome sequencing of ABM was performed with a 20-k library size using a PacBio sequel platform in the Engineering Research Center of the Ministry of Education of Jilin Agricultural University, China (Sossah et al., 2019; Wang et al., 2019). The subreads were assembled using SMARTdenovo^1^. The Core Eukaryotic Genes Mapping Approach (CEGMA) (Parra et al., 2017) and Benchmarking Universal Single-Copy Orthologs (BUSCO) (Waterhouse et al., 2018) were used to test the accuracy and completeness of the assembled genome ABM. The genome sequence of ABM has been submitted to GenBank (accession number: SRHW00000000).

### Genomic Annotation Method of ABM

*De novo* and homologous predictions strategies were used to annotation the three genomes of *A. bisporus*, including the wild strain ABM from the Tibetan Plateau and the reported European strains H97^2^ and H39^3^. The homologous prediction was used with four reference species, including *A. bisporus*, *Coprinopsis cinerea*, *Pleurotus ostreatus*, and *Schizophyllum commune*, and the *de novo* prediction was applied with Augustus, Genescan, GlimmerHMM, and SNAP softwares. Finally, GLEAN^4^ was used to integrate the results obtained by the two methods. The integrated results were used for subsequent analysis: (1) functional annotation of predicted genes was performed with BLAST (*E*-value < 1*e*−5) using public databases including Nr (Non-Redundant Protein Sequence Database), Nt (Nucleotide Sequence Database), Gene Ontology (GO), KOG (Clusters of orthologous groups for eukaryotic complete genomes), Kyoto Encyclopedia of Genes and Genomes (KEGG), Interpro, and SwissProt, etc.; (2) the assembled genome was compared with the repbase database using repetmasker (version 3.3.0^5^) to determine the transposon sequence; the tandem repeats (TRFs) were predicted using TRF^6^ (version 4.04) software, including microsatellites, etc.; (3) tRNAscan-SE software was used for annotation of transfer RNA (tRNA); rRNAmmer software was used as homology prediction and *de novo* prediction of Ribosomal (rRNA); non-coding RNAs such as small nuclear RNA (snRNA) and micro RNA (miRNA) were annotated by the Rfam database.

### Phylogenetic and Evolutionary Analyses

The coding sequences (CDSs) of ABM and nine other reported strains belonging to seven species in NCBI were selected to perform gene family analysis through Orthomcl software (Li et al., 2003), including *A. bisporus* var. *bisporus* H97, *A. bisporus* var. *burnettii* JB137-S8, *Laccaria bicolor*, *C. cinerea*, *Volvariella volvacea*, *S. commune*, *Serpula lacrymans*, and *Coniophora puteana.* The shared single-copy genes were extracted and aligned using Clustal omega software (Sievers and Higgins, 2017). A phylogenetic tree (RAxML 1,000 runs) was constructed using RAxML software (Stamatakis, 2014) with the ML method, and *S. lacrymans* and *C. puteana* were used as the outgroup. The Markov chain Monte Carlo algorithm for Bayes estimation was adopted to estimate the neutral evolutionary rate and species divergence time using the program MCMCTree of the PAML package. The divergence time of *S. lacrymans* and *C. puteana* [70.0–129.4 million years ago (Mya)], *L. bicolor*, and *C. cinerea* (59.3–108.4 Mya), and the divergence time between the two groups (109.89–176.71 Mya) were used as the fossil time correction points (Floudas et al., 2012). The neutral evolution rate of nine strains belonging to eight species and the divergence time of *A. bisporus* strains from different biogeographic distributions were estimated.

The expansion and contraction of gene family were performed using Cafe 3.1 software (De Bie et al., 2006), and a *P*-value of <0.01 was considered to be a gene family with significant expansion contraction. GO and KEGG analyses were performed using Blast2GO software (Conesa et al., 2005) and KEGG Automatic Annotation Server^7^, respectively. For each gene family, the branch locus model in PAML’s codeml tool^8^ was used for positive analysis.

### Whole-Genome Collinearity Analysis of *A. bisporus*

Whole-genome collinearity analysis of ABM, H97, and H39 was performed using Python version of MCscan (JCVI package)^9^ with default parameters. The gff file and cds sequences obtained from the genome annotation were pre-processed to obtain the corresponding input files. The paired syntenic blocks were obtained by comparison, and the unreliable syntenic blocks were filtered out to determine the reliability of the sequences of the two genomes. Linear blocks were used for subsequent mapping.

### Whole-Genome Resequencing of *A. bisporus*

The whole-genome resequencing of 29 *A. bisporus* strains was performed by the sequencing platform of Illumina X-ten with the library size of 350 bp and PE150 strategy at Novogene Co., Ltd. (Beijing, China). The bcl format file for the offline data was converted to the fastaq format using CASAVA software (version 1.8.2; Illumina, Hayward, CA, United States) for subsequent analysis. Clean reads were aligned to the ABM genome using SOAPligner and BWA software (Li et al., 2008, 2009; Li and Durbin, 2009), and the single-nucleotide polymorphisms (SNPs) and insertions–deletions (Indels) were predicted using GATK software (Depristo et al., 2011). The principal component analysis (PCA) of 29 samples was performed using the smartpca program in Eigensoft software (Patterson et al., 2006), and the population genetic structure was analyzed using Structure with 10,000 iterations per run. SNPs of different populations of homologous regions were extracted using SNPhylo pipeline (Lee et al., 2014), and an ML tree was constructed using MEGA software (Kumar et al., 2016).

### Demographic History Analysis of *A. bisporus* Distributed in the Tibetan Plateau in China

We inferred the demographic history of *A. bisporus* using the pairwise sequentially Markovian coalescent (PSMC) model to diploid genome sequences. Based on the resequencing results of 14 wild strains from the Tibetan Plateau in China, SAMtools software (Li et al., 2009) was used to compare BAM files which were used to store sequencing reads alignments information on reference genome, to determine individual genotypes, and low sequencing depths (one-third of average sequencing depth) or high sequencing depths (twofold of average sequencing depth) were masked. The diploid consensus sequence was converted to the desired input format file using fq2psmcfa tool (Kozma et al., 2016). The mutation rate was estimated using the formula: μ = *D* × *g*/2*T*, where *D* is the sequence difference of homolog pair genes between two species, *g* is the estimated generation time, and *T* is the estimated divergence time (Bai et al., 2018). Firstly, genes from two genomes were blast by all-vs.-all manner to identify the best hit homolog gene pairs. Secondly, distmat^10^ was used to calculate Jukes–Cantor distance for those homolog gene pairs (Supplementary Figure S1). Thirdly, the sequence divergence between the ABM and H97 was estimated from the Jukes–Cantor distance distribution. Sequence difference between the ABM and H97 was estimated to be 1.7%, and their divergence time (∼5.5 Mya) was obtained from our result. As a result, the mutation rate (μ) was refined as 1.5 × 10^–9^.

### Genome-Wide Selective Sweep Test in Two Populations of *A. bisporus*

The population from the Tibetan Plateau in China, and Europe and America were considered as two groups, wild strains and cultivated strains. PoPoolation2 software (Kofler et al., 2011) was used to calculate the Fst value of the two populations, VCFtools software (Danecek et al., 2011) was used to calculate the Pi value of the population, and then the combination of Fst and Pi was used to estimate the selective fragments in these two populations. The candidate genes were annotated using the GO and KEGG databases.

## Results

### Genome Sequencing of *A. bisporus* Isolated From the Tibetan Plateau of China

To perform *de novo* whole-genome sequencing of the wild brown strain AB58 from the Tibetan Plateau in China, we isolated the monokaryon ABM using the protoplast-derived method. A total of 8.6 Gb data of ABM were obtained using the PacBio’s sequel platform. The assembled ABM genome was ∼30.4 M with 46.53% GC content, which sequencing depth was ∼282× and consisted of 18 contigs with an N50 of 2.3 Mb (Table 2). CEGMA and BUSCO analyses showed that 96.37% of the core eukaryotic genes and 95.8% single-copy genes were mapped to the ABM genome, respectively. These results indicated that the assembled ABM genome was of high quality.

**TABLE 2:** Summary of genome assembly and annotation of three *A. bisporus* var. *bisporus strains.*

**Species**	**Genome size**	**Scaffold count**	**N50**	**GC content (%)**	**Protein-coding genes**	**Repeat**	**ncRNA**
ABM	30.44M	18	2.3M	46.53	8,562	3.80M (12.50%)	0.11M (0.36%)
H97	30.23M	29	0.3M	46.50	8,631	4.18M (13.83%)	0.77M (2.53%)
H39	30.78M	13	2.5M	46.52	8,492	5.16M (16.76%)	0.02M (0.06%)

### Annotation of *A. bisporus* Collected From the Tibetan Plateau in China

**FIGURE 1:**
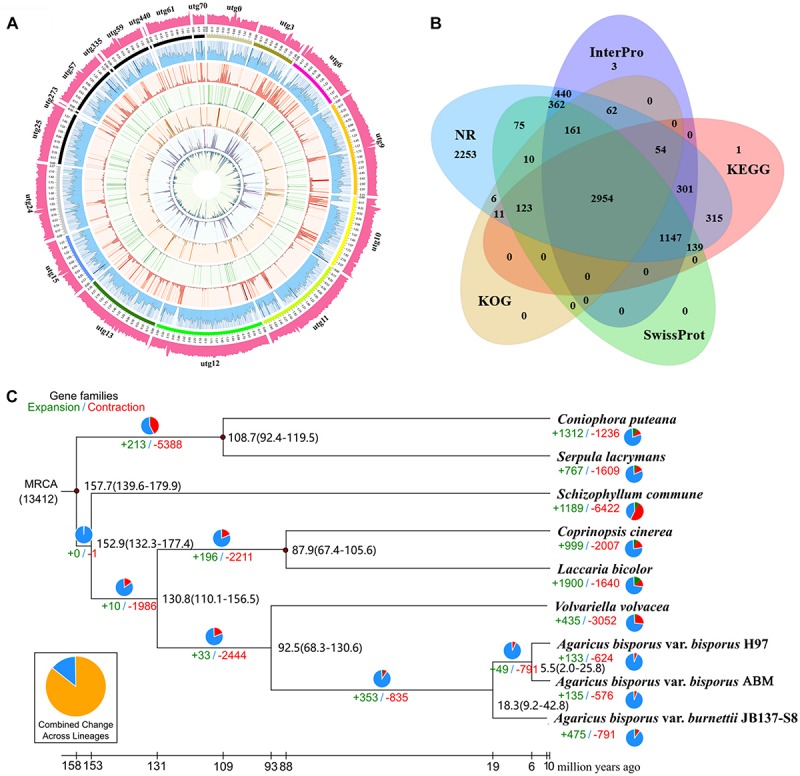
The ABM genome and evolution of *A. bisporus*. **(A)** The *A. bisporus* genome. Outside to inside of concentric circles show GC content, assembly scaffold number, gene density, non-coding RNA (ncRNA), all repeat content, LTR content, LINE content, DNA repeat content, satellite content. **(B)** Venn diagram of the predicted gene number in InterPro, KEGG, SwissProt, KOG, and NR database. **(C)** Numbers on the nodes represent the divergence times. The number of expanded (green) and contracted (red) gene families in each lineage is shown on the corresponding branch. MRCA, most recent common ancestor.

To more accurately predict the protein-coding genes of ABM, we performed homologous and *de novo* annotations. The ABM genome included 8,562 protein-coding genes with an average length of 2,016.5 bp (Figure 1A). The average exon and intron lengths were 268.59 and 115.6 bp, respectively. Among them, 8,483 (99%) genes were annotated by the seven database (Figure 1B), including 8,473 (98.96%), 8,413 (98.26%), 5,484 (64.05%), 5,199 (60.72%), 5,045 (58.92%), 4,971 (58.06%), and 3,381 (39.49%) genes by NCBI Nt and Nr, InterPro, GO, KEGG, Swiss-Prot, and KOG database, respectively.

We identified ∼3.9 M (12.83%) of the repeated sequences in the ABM genome (Figure 1A). Among them, the TRFs and transposable element (TE) sequence length were 0.12 (0.58%) and 3.8 Mb (12.50%), respectively. Among the different TE types, long terminal repeats (LTRs) were most abundant (2.6 Mb), accounting for 8.62% of the genome size, followed by long interspersed nuclear elements (LINEs) (1.32%), transposons (0.60%), and short interspersed elements (SINEs) (0.01%) (Supplementary Table S1). For non-coding RNAs, we identified 201 tRNAs and 170 rRNAs. Among these tRNA, 22 were pseudogenes and the remaining 178 tRNAs were associated with 20 common amino acids. In addition, we predicted 68 miRNAs and 10 snRNAs contained 7 splicing RNAs and 3 C/D nucleolar small RNAs (Supplementary Table S2).

### Molecular Evolution of *A. bisporus* From China and Europe

To study the genome evolution and differentiation of *A. bisporus* from China and Europe, we performed phylogenomic analysis of ABM and eight other reported strains from NCBI. Overall, a total of 1,276 single-copy genes were obtained, which were used to construct the phylogenetic tree (Figure 1C). We found ABM from the Tibetan Plateau in China and H97 from Europe were clustered together as one branch that then clustered with *A. bisporus* var. *burnettii*, which indicated that ABM belonged to *A. bisporus* var. *bisporus*. All these three *A. bisporus* strains then gathered together with *V. volvacea* as a single branch, which were straw rotting mushrooms. We then estimated the divergence time of these nine strains using three correction points of fossil time. The divergence times are presented in Figure 1B, the estimated divergence time between *V. volvacea* and *A. bisporus* was ∼92.5 Mya, between *A. bisporus* var. *burnettii* and var. *bisporus* was ∼18.3 Mya, and between ABM and H97 was ∼5.5 Mya during the Late Miocene. Therefore, *A. bisporus* var. *bisporus* from China and Europe diverged later than *A. bisporus* var. *burnettii*.

### Comparative Genomic Analysis of *A. bisporus*

**FIGURE 2:**
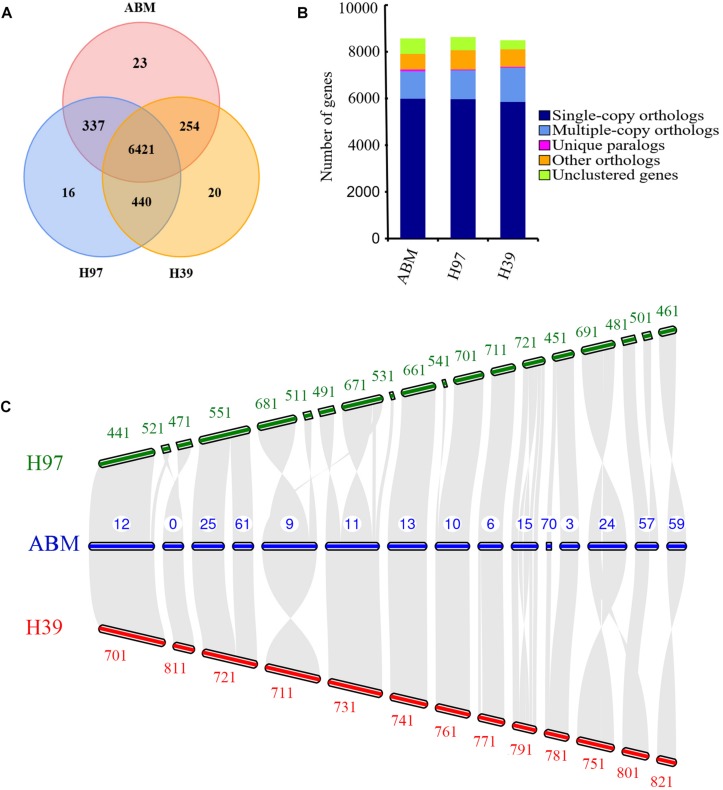
The comparative genomic analysis of three *A. bisporus* strains. **(A)** Unique and share gene families. The number of unique and shared gene families is shown in each of the diagram components. **(B)** Comparison of orthologous genes. **(C)** Whole-genome collinearity analysis, which only showed the contigs exhibiting significant collinear relationship among species.

We analyzed the evolution of gene families in ABM, H97, and H39 of *A. bisporus* var. *bisporus* (Figures 2A,B). We found these three strains shared 6,421 gene families, and contained 23 (ABM), 16 (H97), and 20 (H39) unique gene families (Figure 2A). The ABM-specific gene families mainly included glycosyl hydrolases family 16 (GH16) and genes with the BAG domain and SNM1 domain. We next performed the expansion and contraction of gene families using cafe software (Figure 1C). A total of 133 gene families in ABM underwent expansion during evolution, and of which 34 gene families showed significant expansion. KEGG and GO analyses showed that these expanded gene families were mainly involved in phototransduction and other related metabolic pathways, and associated with transferase activity, phosphorylation, and kinase activity. We also identified 624 contracted gene families with 11 significant contractions, which were mainly involved in the single-organism catabolic process, small molecule catabolic process, organic acid catabolic process, cellular amino acid catabolic process, and carboxylic acid catabolic process. Furthermore, we screened 149 positively selected genes in ABM (*P* < 0.01), which were mainly related to the cellular response to DNA damage stimulus, nucleotide excision repair, mismatch repair, biofilm formation, indole-containing compound biosynthetic process, and tryptophan biosynthesis. These genes might play a crucial role in the regulation of *A. bisporus* in adapting to the harsh Tibetan Plateau environment.

We then conducted the whole-genome collinearity analysis for ABM, H97, and H39 of *A. bisporus* var. *bisporus*, which only showed the contigs exhibiting significant collinear relationship among species (Figure 2C). We found most of contigs of ABM were highly conserved syntenic blocks shared with H97 and H39, including the contig12, contig25, and contig61, contig13, contig10, contig6, contig3 of ABM corresponded to the H97 and H39’s contigs. We also found few contigs showing inversions between ABM and H97, and ABM and H39, such as the contig24 of ABM inversed with the contig691 of H97 and the contig751 of H39. In addition, the contig57 of ABM possessed a block that was inconsistent with any contigs of H39, indicating their loss in the H39 genome. It is worth noting that the obvious inversions between the contig15 of ABM and the contig721 of H97, the contig15 of ABM, and the contig791 of H39. Compared to these two contigs of H97 and H39, the corresponding contig15 of ABM possessed large fragment rearrangements, longer contig length, and more genes (>50). GO and KEGG analyses showed that these 50 genes were mainly associated with oxidase activity and some stress-related pathways such as superoxide dismutase, catalase, and calcium-dependent protein kinases (CDPKs). These differences in ABM, H97, and H39 genomes might play a crucial role in their phenotypic variation and environmental adaptation.

### Genomic Variations in *A. bisporus* Populations

To analyze the population diversity and the genetic differentiation of *A. bisporus*, we carried out the whole-genome resequencing of 29 strains representing the different biogeographic distributions and strain types, including 14 wild strains collected from the Tibetan Plateau in China, 4 Chinese breeding cultivars, and 11 strains from Europe and America. A total of 295 million raw reads were obtained, which resulted in 292 million high-quality clean reads after filtering out the adapters and low-quality data. We selected the ABM genome as the reference genome and found the sequencing depth of each strain was about 35–97× (average 50×) and the average coverage was 95.7%. We then identified a total of 1,052,439 high-quality SNPs and 137,536 InDels in 29 strains. Among these SNPs, 131,967 were non-synonymous SNPs encompassing 8,038 genes, of which 426,311, 187,715, 176,589, and 178,304 were located within exons, introns, upstream, and downstream of the gene, respectively. Among these InDels, 12,916, 25,174, 37,957, and 42,256 were located within exons, introns, upstream, and downstream of the gene, respectively. The variant information could serve as the novel genetic resources for the biology and breeding studies of *A. bisporus*.

### Demographic History of *A. bisporus* Population Distributed in the Tibetan Plateau

**FIGURE 3:**
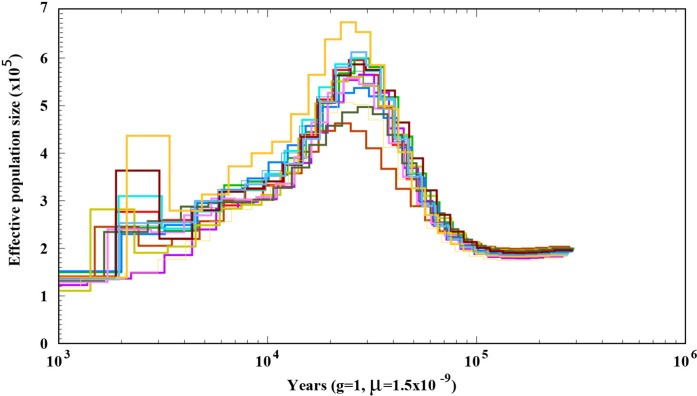
Demographic history of *A. bisporus* distributed in the Tibet Plateau.

To explore the demographic history of *A. bisporus* populations distributed in the Tibetan Plateau, we predicted its effective population size using the PSMC method. We found that its effective population size fluctuated with climate change. When the climate was warm and humid, the effective population size was relatively large. The most significant increase in size occurred during Marine Isotope Stage 3 (MIS3), within the time periods of ∼55–25 ka, and peaked at ∼30–25 ka. The most significant decline in effective population size occurred during the last ice age (∼25–15 ka). Although the last glacial period ended, the temperature remained relatively low, which led to *A. bisporus* populations in the Tibetan plateau undergoing a slow decline (Figure 3).

### Population Structure and Genetic Differentiation of *A. bisporus*

**FIGURE 4:**
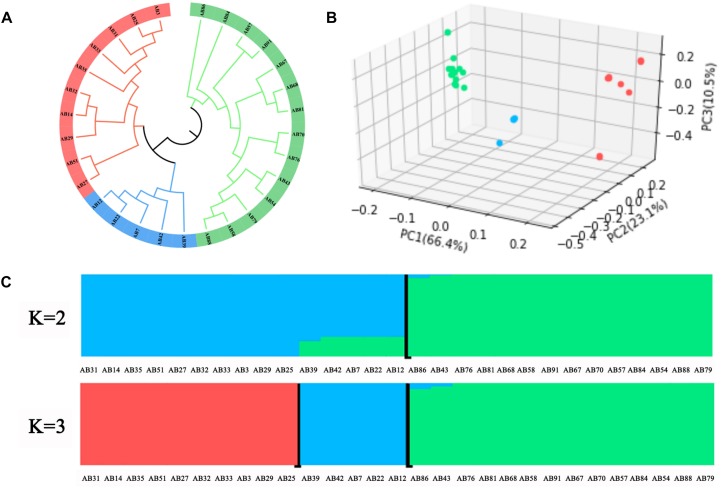
Population genomic analysis of *A. bisporus*. **(A)** Phylogenetic tree. **(B)** PCA plots. **(C)** Genetic structure.

To investigate the phylogenetic relationship of *A. bisporus* germplasm obtained from the Tibetan Plateau in China, Europe, and America, we constructed a phylogenetic tree based on the genome-wide SNPs (Figure 4A). We found the 29 resequencing strains could be clustered into three groups, in which Group I included 14 wild strains from the Tibetan Plateau in China, and Group II included 10 strains from Europe and America breeding cultivars, and Group III included four Chinese and one Europe breeding cultivars. PCA also favored these groupings (Figure 4B). Population structure analysis revealed the division between the Chinese wild strains, and the European and American strains, with little shared ancestry sequences when *K* = 2 (Figure 4C). The population was further diverged at *K* = 3, in which four Chinese breeding cultivars formed a new group containing 70% ancestry sequences of the European and American populations and 30% of the ancestry sequences of the Tibetan Plateau population in China. These results revealed that the Tibetan Plateau population has low introgression with European and American populations at the genome level, and the significant population differentiation between these two populations that likely evolved independently.

### Genome-Wide Selective Sweep Testing of *A. bisporus*

**FIGURE 5:**
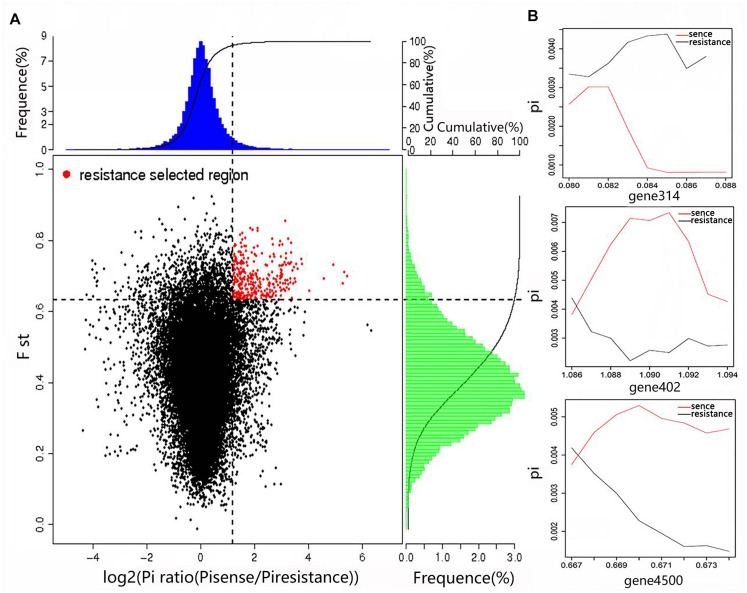
Genomic regions with strong selective sweep signals in the wild high-resistance and cultural high-sense populations of *A. bisporus*. **(A)** Distribution of Pi ratios and FST values. The region of red point were identified as selected regions for two groups. **(B)** Part of selected genes with their genotype diversity.

By combining the Fst value and the θπ ratio, we identified the genomic regions with strong selective signals in the highly resistant Tibetan Plateau population, and the highly sensitive European and American population of *A. bisporus*. A total of 84 candidate genes associated with adaptations to the extreme Tibetan Plateau environment were found among the Tibetan Plateau populations (Figure 5), which were significantly enriched in biological processes such as nitrogen compound metabolism, carbon compound metabolism, cell wall and membrane components, response to external stimulus, signal transaction, DNA repair, and interactions between organisms. We also observed that the circadian and cytochrome genes were also strongly selected in the cultivars of the European and American group, indicating the adaptation of indoor cultivation conditions of the wild strain, and the fruiting body color changed from brown to white by artificial selection.

## Discussion

### Genome Assembly and Annotation of *A. bisporus* Collected From the Tibetan Plateau in China

Wild *A. bisporus* is distributed around the world such as Europe, America, and the Tibetan Plateau in China. We previously found that the Tibetan Plateau wild population possessed higher genetic diversity and high resistance to *Hypomyces perniciosus*, whereas the European and American populations have lower resistance (Fu et al., 2016b). Therefore, we hypothesized that the wild resource in the Tibetan Plateau in China underwent genetic differentiation under the extremely harsh environment. To date, NCBI has published three genomes of the genus *Agaricus*, including *A. bisporus* var. *bisporus* H97, *A. bisporus* var. *bisporus* H39, and *A. bisporus* var. *burnettii* JB137-S8, while without the genome of *A. bisporus* distributed in the Tibetan Plateau in China (Morin et al., 2012; Sonnenberg et al., 2016). Therefore, in this study, we selected the high-quality, brown strain AB58 from the Tibetan Plateau in China to perform whole-genome sequencing and analyzed the genetic differentiation in relation to the European and American populations at the genome level. These studies will provide the genetic background for utilization of germplasms of *A. bisporus* distributed in the Tibetan Plateau.

We obtained the high-quality genome of monokaryon ABM isolated from AB58 from the Tibetan Plateau using the PacBio sequel platform. The genome size of ABM is consistent with H97 and H39 from Europe, whereas *A. bisporus* var. *burnettii* JB137-s8 was ∼2 Mb larger than the three strains. Combined with phylogenetic analysis, we confirmed that the Tibetan Plateau strain ABM and the Europe H97 and H39 are *A. bisporus* var. *bisporus*. To further accurately conduct comparative genomic analysis of different biogeographic strains of *A. bisporus*, we performed re-annotation of H97 and H39 genomes with the same platform and methods used for ABM. A total of 8,631 and 8,492 protein-coding genes were predicted for H97 and H39, respectively, which are similar to the gene number of ABM. However, previous researchers reported that H97 possesses 10,438 protein-coding genes (Morin et al., 2012), and this discrepancy may be attributable to different annotation platforms and methods. Therefore, to avoid systematic biases when conducting comparative genomic analysis, we suggest that researchers perform gene prediction using the same annotation platform and methods for different species or even different strains belonging to the same species.

### Molecular Evolution of *A. bisporus*

Based on the molecular clock method, the estimated divergence time of Tibetan Plateau and European *A. bisporus* was 5.5 (2.0–25.8) Mya. The Tibetan Plateau uplift mainly occurred in 10–8 Mya and began to rapidly uplift in ∼5.3 Mya (Shi et al., 1999a). We hypothesized that the uplift of the Tibetan Plateau might lead to habitat fragmentation of the *A. bisporus* ancestry, which affected the gene flow among the different biogeographic distribution populations. Moreover, these populations in each new distribution might reproduce new genetic mutations that may have been used in adapting to the new environment. Therefore, population differentiation of *A. bisporus* might be due to the long geographical isolation caused by the uplift of the Tibetan Plateau.

As an effective method for demographic history analysis, PMSC has been widely used in many species in recent years (Zhao et al., 2013; Qiu et al., 2015). PMSC analysis indicated that the population size of *A. bisporus* experienced significant expansion and contraction between 55–15 Ka, which was about between MIS5 and MIS2. The climate during this period experienced cold to warm and then to cold, which was consistent with the size fluctuation of *A. bisporus* population from Tibetan Plateau. Therefore, we speculate that the effective population size of Tibetan Plateau *A. bisporus* was affected by environmental changes and climate fluctuations. The most significant expansion of the ancestral population occurred during the MIS3 (∼55–25 ka) (An and Stephen, 1997). It was a warm and humid period in the last glacial period (Shi et al., 1999b), which is suitable for the growth of *A. bisporus*. The most significant decrease in the effective population size occurred ∼25–15 ka during the last ice age. During this period, the climate was extremely cold and dry, and amount of atmospheric dust was 20–25 times more than the present. The ice covered a large area of Tibetan Plateau, which severely threatened the survival of organisms. Therefore, the effective population size of Tibetan Plateau *A. bisporus* began to decrease significantly.

### *Agaricus bisporus* Adaptation to the Harsh Tibetan Plateau Environment

The molecular mechanism of *A. bisporus* adapting to the harsh Tibetan Plateau environment is largely unknown. In this study, we identified the key regulated genes and signaling pathways using comparative genomic and population genomic analysis. Compared to the corresponding contigs in H97 and H39, contig15 of ABM underwent large fragment rearrangements and possessed more genes (>50) that were mainly associated with oxidase activity and calcium-dependent cysteine-type endopeptidase activity. Previous studies have identified oxidoreductases that were associated with fungal stress-resistance by knocking out the superoxide dismutase or catalase genes, leading to a significant reduction in the anti-oxidation and anti-radiation ability of fungi (Jeong et al., 2001; Hwang et al., 2010). Metal-dependent cysteine-type endopeptidase activity may be associated with organisms against pathogens (Hörtensteiner and Feller, 2002; Rodrigo-Moreno et al., 2013), and the gene associated with calcium-dependent cysteine-type endopeptidase activity and located in contig15 of ABM might be related to disease resistance.

We identified specific gene families in ABM, which included GH16 and the gene with the BAG and SNM1 domains. The GH16 gene family is associated with the growth and development of fungi, and plays an important role in drought and other stresses (Zhang et al., 2014; Yuan et al., 2017). The BAG and SNM1 domains are also important domains for stress responses (Williams et al., 2010). We then identified the significantly expanded and contracted gene families in ABM. Among these, we found large-scale expansions of protein kinase and phosphorylation-related gene families such as CDPKs and mitogen-activated protein kinase (MAPK). Previous studies have shown that they play an important role in stress resistance in organisms (Qi et al., 2008; Cai et al., 2014; Sun et al., 2014; Fu et al., 2016a). We also found that the gene families involved in some metabolic processes were contracted in ABM, which might be related to the adaptation of *A. bisporus* to the hypoxic Tibetan Plateau environment. For Tibetan Plateau *A. bisporus*, reducing its own metabolism not only will decrease the demand for oxygen in its metabolic process but also can accumulate organic matter to maintain its growth. In addition, *A. bisporus* grows under strong ultraviolet light and other harsh environmental conditions of Tibetan Plateau, which can significantly damage their DNA. The positively selected genes in ABM related to responses to stimulation, DNA damage repair, and biofilm formation play crucial roles in repairing damaged DNA. Therefore, we provide the genetic evidence that *A. bisporus* has adapted to the Tibetan Plateau environment. In addition, we reveal evidence of the molecular footprints of artificial selection in *A. bisporus* such as the circadian and cytochrome genes.

## Conclusion

In this study, we obtained a high-quality genome sequence of Tibetan Plateau *A. bisporus*. Comparative genomic and population genomic analyses showed that (1) Tibetan Plateau strains belong to *A. bisporus* var. *bisporus*; (2) the divergence time of the Tibetan Plateau and European strains was ∼5.5 Mya, and their differentiation was due to an extended period of biogeographic isolation caused by the uplifting of the Tibetan Plateau; (3) the effective population size of the ancestral Tibetan Plateau population fluctuated with global climate change, and the most significant expansion occurred during MIS3; (4) Tibetan Plateau strains possessed gene families that are associated with adaptation to the harsh Tibetan Plateau environment such as cold, hypoxia, and strong ultraviolet rays; and (5) the Tibetan Plateau population underwent significant divergence from the European and American populations and independently evolved. Therefore, our study highlights valuable genetic resources for the evolution, genetic differentiation, and breeding of *A. bisporus*.

## Data Availability

The genome sequence of ABM has been submitted to GenBank (accession number: SRHW00000000).

## Author Contributions

YF and YL contributed to the conceptualization and funding. LS and YPF contributed to the writing and analysis. YY, XW, WC, and DL contributed to the sample preparation and genome extraction. YHF worked on the Circos software. ZZ and XY contributed to the review and editing of the manuscript.

## Conflict of Interest Statement

The authors declare that the research was conducted in the absence of any commercial or financial relationships that could be construed as a potential conflict of interest.

## Footnotes

1 https://github.com/ruanjue/smartdenovo

2 https://www.ncbi.nlm.nih.gov/genome/858?genome_assembly_id=31528

3 https://www.ncbi.nlm.nih.gov/genome/858?genome_assembly_id=303752

4 http://sourceforge.net/projects/glean-gene

5 http://www.repeatmasker.org/

6 http://tandem.bu.edu/trf/trf.html

7 http://www.genome.jp/tools/kaas/

8 http://abacus.gene.ucl.ac.uk/software/paml.html

9 https://github.com/tanghaibao/jcvi/wiki/MCscan-(Python-version)

10 http://www.bioinformatics.nl/cgi-bin/emboss/distmat
