# Supplementary material for: Genomic Analyses Reveal Evidence of Independent Evolution, Demographic History, and Extreme Environment Adaptation of Tibetan Plateau Agaricus bisporus
Source: Front Microbiol. 2019 Aug 13;10:1786. doi: 10.3389/fmicb.2019.01786 (PMC6700258; doi:10.3389/fmicb.2019.01786)
Supplement: Supplementary file 3 [file Image_1.pdf]

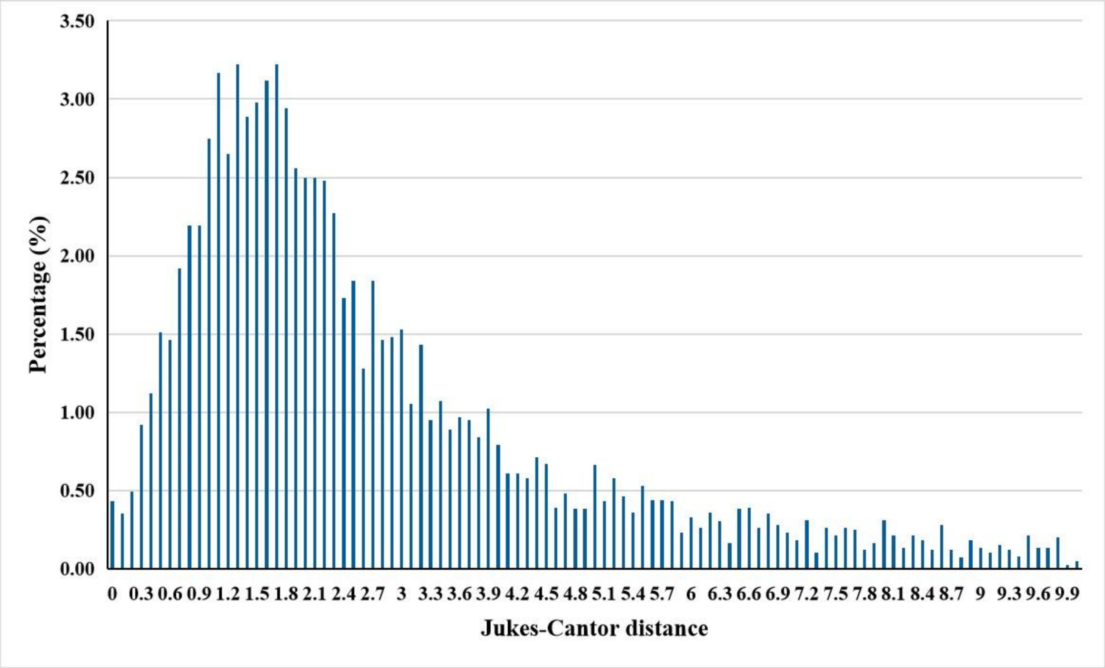

Supplementary Figure S1. Percentage of Jukes-Cantor distance of all the homolog gene pairs.

Additional: Please change the figure legend as “Percentage of Jukes-Cantor distance of all the homolog gene pairs” in line 1137 of proof.
